# Hot Spots of Carbon and Alkalinity Cycling in the Coastal Oceans

**DOI:** 10.1038/s41598-019-41064-w

**Published:** 2019-03-14

**Authors:** Nicholas A. O’Mara, John P. Dunne

**Affiliations:** 1 Department of Earth and Environmental Sciences, Lamont-Doherty Earth Observatory, Columbia University, Palisades, NY USA; 2 Department of Earth, Environmental and Planetary Sciences, Brown University, Providence, RI USA; 3 NOAA Geophysical Fluid Dynamics Laboratory, 201 Forrestal Rd, Princeton, NJ USA

## Abstract

Ocean calcium carbonate (CaCO_3_) production and preservation play a key role in the global carbon cycle. Coastal and continental shelf (neritic) environments account for more than half of global CaCO_3_ accumulation. Previous neritic CaCO_3_ budgets have been limited in both spatial resolution and ability to project responses to environmental change. Here, a 1° spatially explicit budget for neritic CaCO_3_ accumulation is developed. Globally gridded satellite and benthic community area data are used to estimate community CaCO_3_ production. Accumulation rates (PgC yr^−1^) of four neritic environments are calculated: coral reefs/banks (0.084), seagrass-dominated embayments (0.043), and carbonate rich (0.037) and poor (0.0002) shelves. This analysis refines previous neritic CaCO_3_ accumulation estimates (~0.16) and shows almost all coastal carbonate accumulation occurs in the tropics, >50% of coral reef accumulation occurs in the Western Pacific Ocean, and 80% of coral reef, 63% of carbonate shelf, and 58% of bay accumulation occur within three global carbonate hot spots: the Western Pacific Ocean, Eastern Indian Ocean, and Caribbean Sea. These algorithms are amenable for incorporation into Earth System Models that represent open ocean pelagic CaCO_3_ production and deep-sea preservation and assess impacts and feedbacks of environmental change.

## Introduction

Since the industrial revolution, fossil fuel burning and land use change have resulted in significant amounts of carbon dioxide release and global warming^1^. Thus far, about one third of this emitted CO_2_ has been taken up by the ocean^2,3^. This fraction is expected to grow to 90% on millennial timescales^4^. The ocean buffering capacity, or the ability to resist a change in pH, is dependent upon the ocean’s carbon chemistry^5^. Carbonate mineral formation by biological precipitation and preservation within ocean sediment thus represents an important long-term storage mechanism in the global carbon cycle^6^. This process is highly susceptible to rising pCO_2_ and temperatures, and falling ocean pH which increases the solubility of carbonate minerals, reduces biological capacity to produce calcium carbonate (CaCO_3_)^7–11^, and decreases rates of preservation within sediment^12^. While biogeochemically this set of processes serves as a compensating negative feedback increasing the ocean’s ability to buffer anthropogenic CO_2_, it is also expected to have severe deleterious impacts on coastal ecosystems^13^.

Many previous modeling efforts seeking to represent global production and burial of CaCO_3_ have focused on pelagic open ocean production and burial within deep sea sediments, while coastal areas have been largely excluded from global calculations^14–16^. Despite representing less than 7% of the seafloor, coastal and continental shelf (neritic) environments of less than 200 m water depth account for more than half of all CaCO_3_ accumulation in ocean sediment globally^17–19^. It has been suggested that short-term imbalances in neritic carbonate accumulation have caused many of the observed atmospheric carbon dioxide swings in the Pleistocene^20^. Therefore, understanding neritic carbonate budgets is crucially important to understand possible ecological and biogeochemical impacts and feedbacks of global climate change and ocean acidification.

Current estimates of neritic production are based on assumptions about global coverage and production averages of four community types: coral reefs, banks and coastal embayments, carbonate-rich shelves, and carbonate-poor shelves^17–19^. Fluxes within these environments were estimated based on global geochemical constraints, expert opinions, extrapolation to large spatial and temporal averages, and assumptions of the distribution of the four community types. In addition to the question of robustness of these simple empirical estimates, the lack of explicit controlling mechanisms provides no predictive power for estimating changes to these fluxes under different future environmental conditions.

In the present study, we take advantage of new, spatially-resolved physical and biogeochemical datasets to address several of these limitations to estimating the global neritic CaCO_3_ budget. These new datasets include high resolution bathymetric maps, satellite based estimates of pelagic CaCO_3_ fluxes, global maps of CaCO_3_ producing communities including corals and seagrass meadows, and new algorithms for terms in the CaCO_3_ budget. These previously unavailable tools now make possible the spatially explicit characterization of the global neritic CaCO_3_ environment.

## Methods

**Figure 1:**
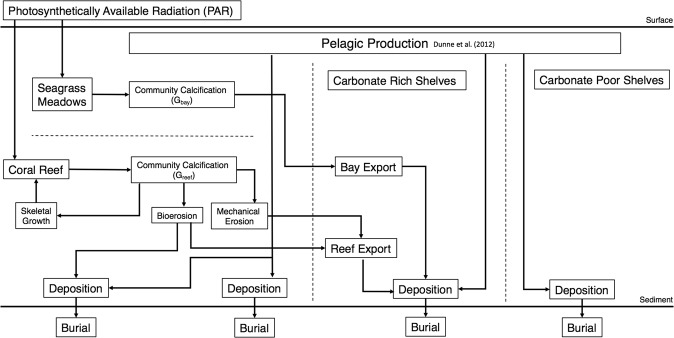
Conceptual model framework depicting the production, accumulation, and transfer of CaCO_3_ within and between the four neritic regions: seagrass meadows, coral reefs, carbonate rich shelves and carbonate poor shelves.

The neritic environment is represented here by a 1° × 1° spatial grid broken down into four community, or region, types: coral reefs, banks/embayments, carbonate-rich shelves, and carbonate-poor shelves for calculation of calcification, deposition and burial rates. In addition to these benthic fluxes, pelagic production estimates calculated by Dunne *et al*.^16^ were extended over the neritic zone. A flow diagram of all of these budget calculations of each cell is provided in Fig. 1.

Globally gridded ocean datasets of annually averaged temperature^21^, salinity^22^, and nutrients^23^ were taken from the World Ocean Atlas 2009. SeaWiFS photosynthetically available radiation (PAR)^24^ and light extinction coefficients^25^ were taken from the NASA Goddard Space Flight Center. Carbon chemistry and pH data were taken from the Carbon Dioxide Information Analysis Center (CDIAC) Global Ocean Data Analysis Project^26^. Surface and bottom water saturation states of calcite and aragonite were calculated according to the United Nations Educational, Scientific and Cultural Organization (1987) algorithm. High resolution bathymetric data were taken from the NOAA Etopo1 Global Relief Model^27^.

Coral reef areas were taken from the United Nations Environmental Programme World Conservation Monitoring Center (UNEP WCMC) GIS map of global coral area which represents global coral reef locations to a resolution of 500 m^28^. Coral area contained within each grid cell was then determined using ArcGIS software (http://desktop.arcgis.com/en/arcmap/) and assuming a constant scaling factor of 30% representing the percentage of live coral cover^29^. Coral CaCO_3_ production by these corals was estimated as a function of sea surface temperature (SST), the saturation state of aragonite (Omega Aragonite), and light availability in the wavelengths photosynthetically available respiration (PAR) scaled by depth in the water column using the mean of the following three algorithms:

Kleypas *et al*.^30^

1 $${E}_{Z}=PAR\ast {e}^{-(K490)(Z)}$$

2 $${G}_{reef1}={G}_{max}\ast tanh(\frac{{E}_{z}}{{E}_{k}})$$

Lough^31^

3 $${G}_{reef2}=0.327\ast (SST)-6.98$$

Silverman *et al*.^32^

4 $$Gi=\frac{24}{1000}(\,-\,0.0177\ast SS{T}^{2}+1.4697\ast SST+14.893)\ast {({\rm{\Omega }}-1)}^{(0.628\ast SST+0.0985)}$$

5 $$\begin{array}{rcl}{G}_{reef3} & = & {k^{\prime} }_{r}\ast Gi\ast {e}^{-{(\frac{{k^{\prime} }_{p}(SST-{T}_{opt})}{{{\rm{\Omega }}}^{2}})}^{2}}\\ {G}_{reef} & = & (\frac{{G}_{reef1}+{G}_{reef2}+{G}_{reef3}}{3})\ast 0.3\end{array}$$

G_reef(1–3)_ is the net community calcification predicted by each model, G_reef_ is the final average net community calcification used here, G_max_ is the maximum calcification given unlimited light, E_k_ is the light (PAR) incident on the surface of the ocean, E_z_ is the light reaching the depth of the coral, z is reef depth in meters, k490 is the light extinction coefficient for light of wavelength 490 nm, SST is the sea surface temperature overlying the coral reef, Gi is the inorganic community calcification, Ω is the saturation state of aragonite, k′_r_ (38 m^2^ m^−2^) and k′_p_ (1 °C^−1^) are scaling coefficients, and T_opt_ is the optimal temperature for growth determined from monthly average values of June in the northern hemisphere and December in the southern hemisphere.

Bioerosion of dead coral materials has been shown to correlate with trophic conditions of the waters the reefs inhabit, low bioerosion when waters are oligotrophic and high bioerosion when water are eutrophic^33–35^.

6 $$Bioerosion=10,\,000\,{e}^{-6{e}^{-5[P{O}_{4}^{3-}]}}$$

Values were thus determined using the above sigmoidal function Eq. (6) where bioerosion is dependent on the concentration of the phosphate ion (PO_4_^3−^) in seawater. Half of this bioeroded material was assumed to remain on the reef while the other half is transported off the reef^35^.

Mechanical erosion is more uncertain than bioerosion and likely varies from reef to reef due to varying amounts of wave action and storm activity in different regions. Here a scalar 10% of G_reef_ was assumed to be removed from the reef due to mechanical erosion^17^. Total export from the reef is the sum of the mechanical erosion and the bioerosion losses.

Total deposition in the coral reef sediment is the sum of the remaining CaCO_3_ from bioerosion and the pelagic flux. Lacking a separate algorithm for the burial efficiency of aragonite, we assumed that aragonite burial efficiency behaved similar to calcite except for modulation via aragonite saturation state. The burial of aragonite from coral bioerosion and calcite from the pelagic flux within the sediment were thus both determined using the Dunne *et al*.^16^ burial algorithm below:

7 $$ \% Burial=\frac{{F}_{btm}-{\Phi }_{R}\ast {F}_{org}}{{(\gamma \ast (1-\Omega +{\Phi }_{org}\ast {F}_{org}))}^{\alpha }\ast {({F}_{lith}+{F}_{btm})}^{\beta }\ast {C}_{0}+{F}_{btm}}$$

F_btm_ is the calcite (aragonite) flux, F_org_ is the organic matter flux, F_lith_ is the terrigenous sediment flux, *Ω* is the saturation state of calcite (aragonite), C_0_ is the density of calcite (aragonite), *γ* is the dissolution rate constant, Φ_R_ and Φ_org_ are dimensionless efficiency terms that affect dissolution at the sediment water interface and the pore water saturation state respectively.

Total accumulation within the coral reef environment is the sum of the burial of pelagic calcite, burial of bioerosion-created aragonite, and skeletal growth of corals.

Coastal embayments were represented here by areas where seagrass meadows were present from the United Nations Environmental Programme World Conservation Monitoring Center (UNEP WCMC) GIS map of global seagrass areas at a resolution of 500 m^36^. Seagrass, or bay area contained within each grid cell was then determined using ArcGIS software (http://desktop.arcgis.com/en/arcmap/).

Seagrass productivity was estimated as a function of SST and PAR in each grid cell where seagrasses were present^37^.

8 $$Productivity=\frac{0.584\ast PAR+16.06\ast SST+285}{2}$$

Estimates of productivity were then used to calculate the total biomass of seagrass present within each grid cell^37^.

9 $$Biomass=\frac{productivity-318.846}{1.712}$$

Net community calcification in bay ecosystems (G_bay_; the sum of epiphyte organisms living on seagrass blades and mollusks living with the seagrass meadows in areas shallower than 30 m) was determined using the following equation^38^:

10 $${G}_{bay}=17.384\ast Biomass+670.26$$

Mechanical erosion was again assumed to remove 10% of community production, like in reef systems, and biological erosion was assumed to export half of the remaining community production out of the seagrass meadow, while the rest is ultimately deposited to the sediment^17,18^. Similar to within coral reef sediments, the Dunne *et al*.^16^ algorithm Eq. (7) was used to calculate burial within the bay sediment. However, the equation was slightly modified to incorporate the additional flux of organic matter from the seagrass blades themselves based on the estimate of Newell and Koch^39^ that organic matter flux in seagrass meadows is approximately 70% from pelagic input.

11 $$ \% Burial\,Bay=\frac{{F}_{btm}-{\Phi }_{R}\ast ({F}_{org}/0.7)}{{(\gamma \ast (1-\Omega +{\Phi }_{org}\ast ({F}_{org}/0.7)))}^{\alpha }\ast {({F}_{lith}+{F}_{btm})}^{\beta }\ast {C}_{0}+{F}_{btm}}$$

Unlike in the case of corals where we assume long term accumulation (burial) in skeletons, for seagrass bays we assume accumulation is equal to the burial within sediments because blade lifespans shorter than one year limit long-term storage of CaCO_3_ within the living organisms^40^.

Carbonate-rich shelves are defined as the non-seagrass and non-coral areas shallower than 200 m within grid cells that contain either seagrasses or corals. Deposition of CaCO_3_ is equal to export from either or both of those two communities spread evenly over the area of the grid cell not occupied by those communities in addition to the pelagic flux. Burial within carbonate-rich shelves are then determined using Eq. (7). Carbonate-poor shelves are defined as grid cells that contain shelf areas (<200 m) that do not contain any coral reefs or seagrass bays and the only CaCO_3_ input is the pelagic flux. Burial is then calculated using Eq. (7).

Uncertainty in these budget estimates was determined using a Monte Carlo approach where each variable was allowed to vary uniformly by up to 50%. The distribution for each variable in the analysis was randomly sampled and carbonate accumulation rates were recalculated (n = 10,000) to estimate uncertainty in the CaCO_3_ accumulation rate in each neritic region. The standard deviation of the resulting distributions (±1σ error) are shown in Table 1. While the total carbonate accumulation from the pelagic flux (178%) and carbonate-poor shelves (85%) remain quite uncertain, the uncertainty in the coral reefs (46%), carbonate-rich shelves (39%), and the bays (46%) are all less than the 50% variability introduced here suggesting that these uncertainties are only a product of the uncertainty in the underlying data that constrain this analysis. The total benthic accumulation is especially resilient to changes in the model parameters (31%).

**Table 1:** CaCO_3_ benthic and pelagic flux and accumulation estimates for neritic regions from model output (this study) and previous estimates (Iglesias-Rodriguez *et al*.^19^).

Neritic Region	Area (10^12^ m^2^)		Flux (g C m^−2^ yr^−1^)		Accumulation (Pg C yr^−1^)		Uncertainty	
	Iglesias-Rodriguez *et al*. (2002)	This Study	Iglesias-Rodriguez *et al*. (2002)	This Study	Iglesias-Rodriguez *et al*. (2002)	This Study	Iglesias-Rodriguez *et al*. (2002)	This Study (1σ)
Coral Reefs	0.6	0.25	140	334	0.084	0.084	±50%	±46%
Carbonate Shelves	10	7.30	3.8	5.34	0.038	0.037	>100%	±39%
Bays	0.8	0.34	30	125	0.024	0.043	±100%	±46%
Carbonate Poor Shelves	15	16.49	0.8	0.012	0.012	0.0002	>100%	±178%
Pelagic	—	24.38	—	0.012	—	0.0013	±100%	±85%
Benthic	26.4	24.38	—	—	0.158	0.163	±100%	±31%
Total					0.158	0.164		

## Results

**Figure 2:**
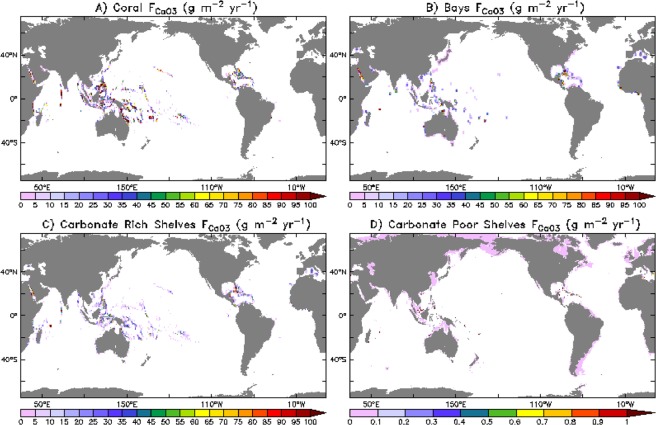
Global maps of model derived carbonate burial fluxes in (**A**) coral reefs, (**B**) seagrass bays, (**C**) carbonate rich shelves, and (**D**) carbonate poor shelves generated using the data analysis tool Ferret (v7) (http://ferret.pmel.noaa.gov/Ferret).

The outputs of the described model framework represented spatially in Fig. 2 as well as values of the previous neritic carbonate budget estimate Iglesias-Rodriguez *et al*.^19^ are summarized in Table 1.

We find total accumulation within the neritic zone study (0.164 Pg C yr^−1^) similar to the Iglesias-Rodriguez *et al*.^19^ estimate (0.158 Pg C yr^−1^). Additionally, the pelagic contribution to neritic CaCO3 accumulation (0.0013 Pg C yr^−1^) is much smaller than the benthic contribution (0.163 Pg C yr^−1^). The calculated accumulation within coral reef communities is approximately 0.084 (Pg C yr^−1^) and is actually identical to the prediction of the Iglesias-Rodriguez *et al*.^19^ estimate despite varying estimates of coral reef area and fluxes of CaCO_3_. Likewise, we find very similar values for carbonate-rich shelf accumulation and flux rates despite slightly different total region areas. However, we find much higher flux and accumulation rates in bay sediment despite a smaller region areas and much lower carbonate-poor shelf flux and accumulation rates despite a larger region area than do Iglesias-Rodriguez *et al*.^19^.

As expected from their physiology, over 99% of warm water coral reef calcification is meridionally restricted to the tropics, and over half (53%) is restricted to 1/6th of the planet in the Western Tropical Pacific, from 120°E to the date line. The area west of 120°E into the Eastern Indian ocean at 90°E accounts for another 16%, and the Caribbean another 11%. Less expected, the distribution of both carbonate rich shelves (96%) and bays (93%) are also found to be restricted into the tropics (30°N–30°S). Burial of these CaCO_3_ regimes are more broadly distributed across the tropical coastal regions with only 37% shelf and 28% bay burial in the Western Pacific, and the Caribbean accounting for a full 20% of bay burial (Table 2).

**Table 2:** Regional % contribution of the Western Pacific Ocean, Eastern Indian Ocean/Oceania, and the Caribbean Sea to the total accumulation of CaCO_3_ in coral reefs, carbonate shelves, and bays determined from model output.

Neritic Region	Total Accumulation (Pg C yr^−1^)	Total Area (10^12^ m^2^)	Western Pacific (120°E–180°) %	Eastern Indian/Oceania (90°E–120°E) %	Caribbean (90°W–60°W, 8°N–31°N) %
Coral Reefs	0.084	0.25	53	16	11
Carbonate Shelves	0.037	7.30	37	9	17
Bay	0.043	0.34	28	10	20

**Figure 3:**
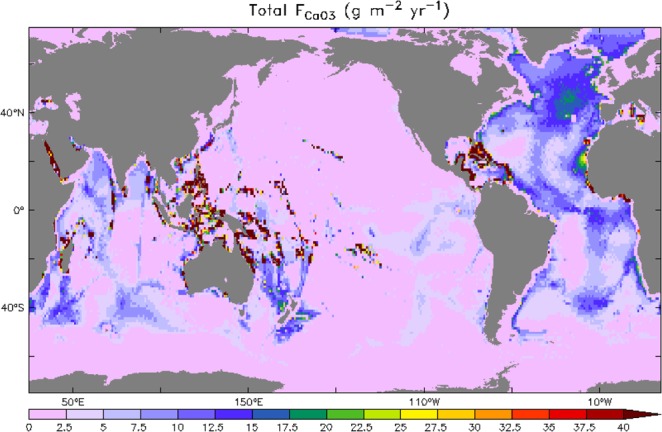
Global map of seafloor carbonate burial flux including both the neritic zone (this study) and the deep sea (Dunne *et al*.^16^) generated using the data analysis tool Ferret (v7) (http://ferret.pmel.noaa.gov/Ferret).

By extending past spatially explicit analysis into neritic regions, this study completes the global, spatially explicit picture of neritic and pelagic CaCO_3_. Figure 3 shows these neritic CaCO_3_ burial fluxes determined here plotted along with previous estimates for the pelagic ocean^16^. The relatively intense local burial of CaCO_3_ in neritic regions and the regionally focused burial in the Western Tropical Pacific and Atlantic Oceans illustrate the potential for neritic burial to influence both the meridional and interbasin structure in CaCO_3_ burial. While pelagic burial is dominated by the North Atlantic, neritic burial is dominated by the tropics with the Western Tropical Pacific accounting for half of the global total.

## Discussion

The Iglesias-Rodriguez *et al*.^19^ CaCO_3_ budget estimate of coral reef accumulation relied heavily upon assumptions made on the spatial extent, production, and burial rates within coral communities globally. Many of these assumptions are carried over from previous carbonate budget analyses^17,18^, which due to limited measurements of locations of coral reefs assumed corals inhabited all environments suitable for growth, resulting in over estimations of total global coral areas 0.6 × 10^12^ m^2^ compared to modern maps 0.25 × 10^12^ m^2^. Despite this over estimation of spatial extent, spurious Holocene accumulation averages, assumptions about large reductions of calcification in lagoon areas, and difficulty in measuring coral skeletal growth resulted in underestimations of total annual accumulation rates.

Carbonate-rich shelf environment estimates predicted by this model agree well with the Iglesias-Rodriguez *et al*.^19^ estimates which provides increased confidence in the magnitude of these fluxes on the global scale. More importantly, this good comparison provides us with the additional opportunity to leverage the geographically explicit nature of our dataset and resolve region specific fluxes beyond a simple global budget and highlight ‘hot spots’ in the carbon cycle. The lower global area here is compensated by a higher average area-specific flux. In contrast, the model developed here predicts much higher accumulation rates of calcium carbonate within coastal embayment regions, or seagrass meadows, than previous estimates. Initial estimates of bank and bay areas were defined as regions shallower than 50 m and 30 m respectively that are partially surrounded by land, and assumed to be dominated by benthic algae rather than corals^17^. This resulted in a large over estimation of total bay area (0.8 × 10^12^ m^2^) in combination with limited sampling and reliance on long-term average deposition rates resulted in underestimates of deposition within these environments. This study takes a more specific approach in defining embayments as only those enclosed areas containing benthic seagrass communities.

A recent literature review^41^ of global seagrass meadow CaCO_3_ burial, estimated that seagrass meadows have a range between 0.177–0.6 × 10^12^ m^2^ and an accumulation rate of 0.022–0.076 Pg C yr^−1^ and a flux rate of 126.3 ± 0.7 gC m^−2^ y^−1^. The total area (0.34 × 10^12^ m^2^), accumulation rate (0.043 Pg C yr^−1^), and flux values (125 gC m^−2^ y^−1^) predicted by this model agree well with this study, which give credibility to the present estimates over previous attempts.

Carbonate-poor shelves have been predicted^19^ to accumulate as much as 0.012 Pg C yr^−1^. The present study predicts a vastly smaller annual accumulation of 0.0002 Pg C yr^−1^. Production rates of carbonate are based on the dubious assumption that production in the sediment must exceed surface pelagic production^18^. This assumption allowed for carbonate-poor shelves to balance the biogeochemical gap between the measured fluvial (1.3 Tg C yr^−1^)^42^ and estimated hydrothermal (0.3 Tg C yr^−1^)^43^ and groundwater (0.5 Tg C yr^−1^)^18^ inputs with total neritic accumulation, i.e. the estimates of coral reefs, bays, and carbonate-rich shelves^18^. This model, however, assumes benthic production of carbonate in carbonate-poor shelves equals pelagic input. The present study shows that larger predicted fluxes within coastal bay environments make this budget-based carbonate-poor shelf production assumption unnecessary as the flux is instead estimated explicitly.

Complementary to information about global CaCO_3_ production in various neritic zone environments, this analysis has further revealed three key coastal areas, or hot spots, in the Western Pacific Ocean, Eastern Indian Ocean, and Caribbean Sea that together represent more than half of the global coastal carbonate burial flux. The ‘hot spots’ the small coastal areas in these three geographic regions represent important focal points in the global coastal carbonate budget and thus warrant increased study and protective measures to mitigate large-scale anthropogenic perturbations to global carbonate budget.

The Iglesias-Rodriguez *et al*.^19^ budget approximated uncertainties on the order of 50 to >100%; here we find similar errors ranging 31 to 178%. However, these new estimates have the added benefit of coming from a quantitatively defined model framework and thus represent a refinement of previous qualitative estimates. Furthermore, we demonstrate significant reductions in uncertainty estimates of carbonate rich shelves, bays, and total benthic accumulation. Unfortunately, our ability to ground-truth estimates for the CaCO_3_ budget determined here is limited by the paucity of relevant independent observational data. In contrast to the more spatially homogenous deep sea sediments, the heterogeneity of coastal carbonate producing ecosystems makes it difficult to extrapolate from single point measurements to global or even regional accumulation rates. Future sampling will allow for local comparisons between observed accumulation rates and predictions from this model. Despite the uncertainty of our results, this approach represents a carefully constructed null hypothesis for the role of carbonate producing ecosystems in the coastal ocean alkalinity budget.

There are several aspects of this study that warrant further investigation. Foremost among these would be to improve both the representation of heterogeneity at the local spatial scale within these environments and the mechanistic representation of these calcite and aragonite producing ecosystems to better capture their potential vulnerability to dissolution with enhanced ocean acidification. For example, live coral cover percentages, assumed here to be 30%, and mechanical erosion rates of reefs, assumed here to be 10% of net calcification, are in reality regionally variable. This study assumed simple, globally applicable production rates for coral, seagrass, and shellfish communities that are, in reality, likely highly variable. Furthermore, it does not account for the potential role of direct human actions such as pollution, habitat destruction, and fishing that are also known to have deleterious impacts^44,45^. Finally, while this model can project the potential response of the global neritic carbonate cycle, it cannot resolve the role of local heterogeneity in habitat and productivity in modulating sensitivity of carbonate producing ecosystems to environmental stressors. Such deficiencies in the current approach should be addressed with targeted and comprehensive process studies in these dynamic coastal environments.

## Conclusions

In this study, we address several deficiencies with previous estimates of neritic CaCO_3_ accumulation by (1) incorporating explicitly measured areas of neritic community types rather than hypothetical/potential areas, (2) spatially explicit rates of CaCO_3_ production and burial from an expanded observational database, (3) improved regional information and predictive power about future alterations to CaCO_3_ accumulation as a result of environmental change.

In its spatially explicit and comprehensive synthesis of the neritic CaCO_3_ budget, this study improves upon past estimates and creates a set of parameterizations that predict fluxes of CaCO_3_ within the neritic zone using updated global maps of community areas and environmental constraints to estimate CaCO_3_ accumulation rates. Model output (1) confirms prediction of dominance of benthic over pelagic production in the neritic zone, (2) corroborates current total neritic CaCO_3_ accumulation with coral reefs contributing approximately half of the total and carbonate rich bays and shelves each contributing about a quarter, but predicts lower and higher rates in carbonate-poor shelves and embayments respectively, (3) despite only representing only 7% of the seafloor, neritic environments account for more than half of the total ocean annual CaCO_3_ burial flux and almost entirely restricted to the tropical oceans with more than half of coral burial contained within the Western Tropical Pacific and total coastal burial contained within three hot spots, and (4) is readily amenable to implementation in global earth system models of carbon cycle impacts and feedbacks on ocean carbon uptake under environmental change. Future research is needed on the role of local heterogeneity in habitat, productivity, and human activity in modulating sensitivity of carbonate producing ecosystems to environmental stressors to improve regional and global scale predictions of how ocean acidification will influence carbonate-producing ecosystems.

## Supplementary information

Dataset 1

## Data Availability

All datasets used for this analysis are available from their respective intext citations. The resulting carbonate flux data generated by this study can be found in the Supplementary Data for this article.
