# Author Correction: Hot Spots of Carbon and Alkalinity Cycling in the Coastal Oceans

**DOI:** 10.1038/s41598-020-65546-4

**Published:** 2020-05-20

**Authors:** Nicholas A. O’Mara, John P. Dunne

**Affiliations:** 1 Department of Earth and Environmental Sciences, Lamont-Doherty Earth Observatory, Columbia University, Palisades, NY USA; 2 Department of Earth, Environmental and Planetary Sciences, Brown University, Providence, RI USA; 3 NOAA Geophysical Fluid Dynamics Laboratory, 201 Forrestal Rd, Princeton, NJ USA

Correction to: *Scientific Reports* 10.1038/s41598-019-41064-w, published online 14 March 2019

The Supplementary Information file originally published with this Article was corrupted. This error has been corrected in the Supplementary Information file that now accompanies the Article.

